# Natural History and Microbiological Profiles of Patients With Acute Pancreatitis With Suspected Infected Pancreatic Necrosis

**DOI:** 10.7759/cureus.71853

**Published:** 2024-10-19

**Authors:** Praveen Kumar Loganathan, Gaurav Muktesh, Rakesh Kochhar, Jayanta Samanta, Jimil Shah, Archana Angrup

**Affiliations:** 1 Gastroenterology, Postgraduate Institute of Medical Education and Research, Chandigarh, IND; 2 Medical Microbiology, Postgraduate Institute of Medical Education and Research, Chandigarh, IND

**Keywords:** acute pancreatitis, extrapancreatic infection, gram-negative bacteria, infected pancreatic necrosis, microbiological profile in pancreatitis, multi-drug resistance, organ failure

## Abstract

Introduction: Acute pancreatitis (AP) is a prevalent emergency. The clinical spectrum of the condition is varied, ranging from a mild to a malignant course with higher mortality rates. Infection of pancreatic/peri necrosis, extrapancreatic infections, and organ failure are significant complications in AP. In the recent era, microbiological composition has shifted more towards multi-drug-resistant organisms due to irrational antibiotic use.

Objective: This study aims to understand the natural history of patients with suspected infected pancreatic necrosis (IPN), the prevalence of multi-drug-resistant organisms (MDROs), and their antibiotic susceptibility patterns.

Methods: This prospective, observational study was performed on 130 cases of acute necrotizing pancreatitis (ANP) with suspected IPN that were evaluated during their period of admission in the department of gastroenterology, emergency medicine, or surgery ward of the Postgraduate Institute of Medical Education and Research, Chandigarh, India, a tertiary hospital. The details of outcomes were recorded. The organisms in cases with IPN and antibiotic resistance patterns of various organisms were studied.

Results: The most prevalent site of necrosis was combined pancreatic and peripancreatic tissues in 111 (85.4%) patients. The computed tomography severity index (CTSI) had a mean of 8.72±1.43. Of all participants, 36 (27.7%) patients had <30% necrosis, 47 (36.2%) had 30%-50% necrosis, and 47 (36.2%) had >50% necrosis. Overall, 80 (61.5%) patients had organ failure, with acute lung injury (ALI) being the most common. Rates of complications were markedly greater in cases with higher pancreatic necrosis and with infected necrosis (p<0.05). The most common organism isolated from necrotic tissue was *Escherichia coli* (*E. coli*) in 17 (32.07%) cases, followed by *Acinetobacter baumannii *(*A. baumannii*) in 12 (22.64%) cases. The most common extrapancreatic infection site was respiratory tract infections. *Pseudomonas aeruginosa* (*P. aeruginosa*) and *A. baumannii* revealed maximum resistance to most of the drugs.

Conclusion: Infected pancreatic necrosis is linked to high rates of morbidity and mortality. Our study suggests that irrational usage of antibiotics increases the incidence of combined infection, especially with higher rates of multi-drug-resistant infections.

## Introduction

Acute pancreatitis (AP) is an inflammatory disease of the exocrine part of the pancreas that results from unregulated stimulation of trypsin within pancreatic acinar cells [1]. It is one of the more frequent gastroenterological emergencies, with incidence ranging from five to 73 per 100,000 per year [2]. Necrotizing pancreatitis occurs when the damage from pancreatitis causes tissue in the pancreas to die or necrotize and is defined radiologically as the non-enhancing areas of pancreatic parenchyma on contrast-enhanced computed tomography (CECT) of the abdomen, which is observed in about 5%-10% of cases with AP [3]. Acute necrotizing pancreatitis (ANP) can be either pancreatic, peripancreatic, or combined peripancreatic and pancreatic necrosis [3]. Necrosis can stay sterile or become infected. Infection of pancreatic necrosis increases the risk of mortality by up to 30% [4,5]. When percutaneous, image-guided fine needle aspiration (FNA) yields positive results for bacteria and/or fungus on Gram stain and culture, or when there is extraluminal gas in the pancreas and/or peripancreatic tissues on CECT, it is assumed that an infection is present. The microbiological composition of the necrotic pancreas consists mainly of Gram-negative bacteria, which mostly originate from the gastrointestinal tract (GIT) by translocation from the gut. Recently, it has been shown that the most frequent microorganisms isolated from cases with AP with infected pancreatic necrosis (IPN) are *Escherichia coli* (*E. coli*), *Klebsiella pneumonia* (*K. pneumonia*), and *Acinetobacter baumannii *(*A. baumannii*) [6]. 

Compared to patients with sterile necrosis, patients with infected necrosis had a higher risk of organ failure and ICU admission [4]. In cases with infected necrosis, there is an increased risk for multiple interventions and the need for surgery. In general, cases with infected necrosis are more prone to morbidities and mortality compared to patients with sterile necrosis [5].

Due to injudicious use of antibiotics, the incidence of multi-drug resistance (MDR) strains is increasing [7]. Moreover, the recent trends in antibiotic resistance patterns and the prevalence of MDR strains in patients with AP with IPN are unknown. Hence, we aimed to assess the natural history of patients with suspected IPN, their antibiotic susceptibility patterns, and their microbiological profile in a tertiary care academic centre.

## Materials and methods

Study protocol

This was a prospective observational single-centre study investigating the natural history and microbiological profile along with the drug resistance patterns of patients with suspected IPN. We selected all individuals aged more than 18 years old with suspected IPN from the medicine emergency unit, gastroenterology ward, gastroenterology ICU, or surgery ward of the Postgraduate Institute of Medical Education and Research, Chandigarh, India, a tertiary care hospital in India during the period from January 2021 to December 2021. We excluded patients <18 years of age, patients with HIV infection, patients with chronic pancreatitis/acute on top of chronic pancreatitis, and those who refused to give consent. Infected pancreatic necrosis was suspected "when a patient with necrotizing pancreatitis developed a prolonged high temperature of >38.0°C after the first week of illness without any other focus of sepsis along with leucocytosis and decline in clinical condition" [8]. The research protocol was reviewed and permitted by the institutional research and ethics committee (approval number: NK/6218/MD/725). After participants were adequately briefed on the study's goals, their written informed consent was obtained. The subject was free to withdraw from the study at any moment, and participation was entirely voluntary. According to the Declaration of Helsinki, all steps of data collection, entry, and analysis were conducted in a highly confidential and private manner.

Sample collection and processing

Body fluid samples were sent for bacterial and fungal cultures. These samples included percutaneous catheter drainage (PCD) fluid from the pancreatic collection, blood, urine, sputum, pleural fluid, ascitic fluid, and endotracheal tube (ET) aspirate in the case of intubated patients. Until the sepsis subsided or the cause of the sepsis was identified, blood cultures for bacteria and fungi were performed twice a week. Percutaneous aspiration was used to get body fluids from sterile locations at the peritoneal or pleural levels.

Management (standard medical therapy)

**Figure 1:**
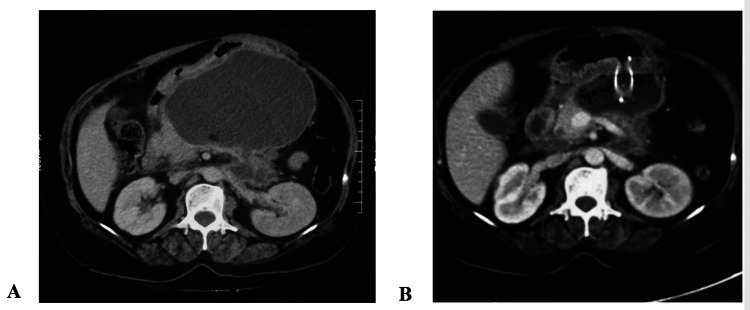
A: Contrast-enhanced computed tomography showing large infected walled-off necrosis in lesser sac compressing stomach; B: Contrast-enhanced computed tomography of the same patient after 72 hours of drainage showing significant reduction in size of walled-off necrosis after endoscopic drainage with metal stent

For all the enrolled patients, initial clinical assessment and risk stratification were done. All the clinical parameters, including the presence of pain, fever, and other symptoms, and basic laboratory parameters (hemogram, amylase, serum glucose, biochemistry, and coagulogram), along with the chest X-ray (CXR), were recorded. The severity of pancreatitis and the existence of organ failure were assessed based on the modified Marshal scoring system for organ failure [9]. Also, scores of the Bedside Index for Severity in Acute Pancreatitis (BISAP) [10] and Acute Physiology and Chronic Health Examination (APACHE) II [11] were calculated. Analgesics, oxygen, IV fluids, and nutritional management were given as per the requirement. Aggressive hydration (250-500 ml per hour) with crystalloids was given to all patients unless precluded by comorbidity. A CECT of the abdomen was done after five to seven days of the onset of pain in patients who failed to improve clinically or in whom pancreatic necrosis was suspected (Figure 1). Computed tomography severity index (CTSI) and percentage of necrosis were analyzed in all patients. 

Empirical antibiotics were initiated in cases with suspected infection and subsequently upgraded according to sensitivity reports.

Every detail of the results was documented, such as the length of hospital stay, the requirement for an intensive care unit stay, organ failure, the requirement for percutaneous, endoscopic, or surgical procedures, and death. The organisms in patients with IPN and antibiotic resistance patterns of various organisms were studied.

Statistical analysis 

Data were compiled in a Microsoft Excel sheet (Microsoft Corp., Redmond, WA) for all the variables of interest. The compiled data were presented in the form of tables and graphs. The statistical analysis was carried out using SPSS for Windows, version 15.0 (SPSS Inc., Chicago, IL). Quantitative variables were summarized in the form of mean±SD or median±median±interquartile range (IQR) as applicable. Qualitative variables were summarized in the form of proportions or percentages. Chi-squared tests were used to compare differences among the two groups for the qualitative data, and if there was a significant difference, further Bonferroni correction methods were applied. Independent samples t-test and Mann-Whitney U tests are used to compare differences among a categorical and a continuous variable. All statistical tests were performed at a 5% level of significance (α = 0.05), i.e., a p-value <0.05 was considered significant.

## Results

Our work included a total of 130 cases that fulfilled our inclusion criteria with suspected IPN. The patients were between the ages of 20 and 74, with a mean age of 39.75±12.68 years. There were 95 males (73.1%) and 35 females (26.9%) in the study group.

The most common cause of AP among our cases was alcohol consumption in 57 (43.8%) patients, followed by gallstone disease in 34 (26.2%) patients. Other less common aetiologies were a combination of alcohol and glycogen storage disease (GSD) in 11 (8.5%) patients, post-endoscopic retrograde cholangiopancreatography (ERCP) pancreatitis in three (2.3%) patients, hypertriglyceridemia in three (2.3%) patients, and post-traumatic pancreatitis in one (0.8%) patient. However, aetiology could not be found in 21 (16.2%) cases and was considered idiopathic AP.

**Figure 2:**
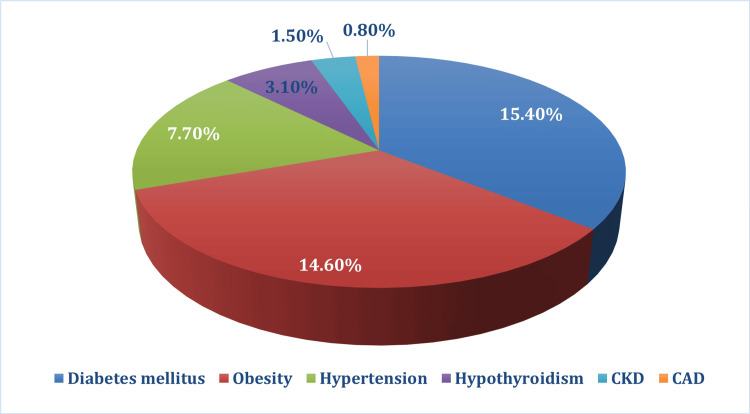
Distribution of comorbidities among study participants CKD: chronic kidney disease; CAD: coronary artery disease

Among the 130 patients in the study, 37 (28.5%) cases had one or more comorbidities. Diabetes diagnosed in 20 (15.4%) cases and obesity in 19 (14.6%) cases were the most common comorbidities, as shown in Figure 2.

Fever was the most common symptom of patients with suspected IPN at presentation, found in 112 (86.2%) patients. The mean±SD baseline haemoglobin level of the study group was 10.65±2.17 (g/dl). Laboratory analysis revealed high mean/median levels of total leucocyte count (TLC), urea, creatinine, and total bilirubin with low levels of albumin (Table 1).

**Table 1:** Clinical and laboratory variables of cases with suspected IPN (N=130) ALP: alkaline phosphatase; ALT: alanine aminotransferase; APACHE: Acute Physiology and Chronic Health Evaluation Score; AST: aspartate aminotransferase; BISAP: Bedside Index of Severity of Acute Pancreatitis; FiO2: fraction of inspired oxygen; IPN: infected pancreatic necrosis; iPTH: intact parathyroid hormone; PTH: parathormone; PaO2: partial pressure of oxygen; TLC: total leucocyte count; SIRS: systemic inflammatory response syndrome

Parameters	N (%), Mean±SD, Median (IQR)
Clinical parameters	
Abdominal pain, n (%)	102 (81.5%)
Fever, n (%)	112 (86.2%)
Dyspnea, n (%)	61 (46.9%)
SIRS at admission, Mean±SD	2.79±1.40
PaO2/FiO2 ratio at admission, Mean±SD	331±103
BISAP score, Mean±SD	2.480±0.5986
APACHE II score, Mean±SD	9.582±5.446
Modified Marshall score, Mean±SD	4.92±1.88
Laboratory parameters	
Haemoglobin (g/dl), Mean±SD	10.65±2.17
TLC (µL), Mean±SD	17996.00±5067.59
Platelet(x10^9^/L), Mean±SD	254.01±90.83
Urea (mg/dl), Median (IQR)	42.8 (28.65-56.95)
Creatinine (mg/dl), Median (IQR)	2.08 (1.36-3.21)
Total bilirubin (mg/dl), Median (IQR)	1.99 (0.76-3.02)
Conjugated bilirubin mg/dl), Median (IQR)	1.27 (0.58-2.19)
AST (U/L), Median (IQR)	88.25 (47.5-128.95)
ALT (U/L), Median (IQR)	97.42 (51.12-144.76)
ALP (U/L), Median (IQR)	198.76 (98.5-306.45)
Total protein (g/dl), Mean±SD	6.50±2.73
Albumin (g/dl), Mean±SD	2.97±0.67
Calcium (mg/dl), Mean±SD	9.26±2.89
Vitamin D (ng/ml), Mean±SD	15.15±6.04
iPTH (pg/ml), Median (IQR)	58.4 (20.5-68.3)
Triglycerides (mg/dl), Mean±SD	178.22±43.50

In our study group, 85 (65.4%) cases were referred from other hospitals, and 45 (34.6%) cases were directly admitted. Among all study participants, only three (2.3%) patients were admitted to our institute on the same day of illness, and 37 (28.5%) patients were admitted during the first seven days, while the majority of cases, 90 (69.1%) patients, were admitted after the first week of illness.

Regarding radiological investigations, a CECT scan of the abdomen was done in all patients with suspected IPN. The most common site of necrosis was combined pancreatic and extrapancreatic tissues in 111 (85.4%) patients. Extrapancreatic necrosis alone was observed in 16 (12.3%) cases, and necrosis of pancreatic parenchyma alone was present in 3 (2.3%) cases. The computed tomography severity index (CTSI) had a mean of 8.72±1.43.

Regarding complications, 51 (39.2%) patients had vascular complications. Of these complications, 19 (37.25%) had portal vein thrombosis (PVT), 12 (23.52%) had splenic vein thrombosis (SVT), 10 (19.6%) had superior mesenteric vein thrombosis (SMVT), one (1.96%) had left gastric artery pseudoaneurysm, and nine (17.6%) had more than one vessel thrombosis.

Of all our participants, 36 (27.7%) patients had <30% necrosis, 47 (36.2%) had 30%-50% necrosis, and 47 (36.2%) cases had >50% necrosis.

Overall, 80 (61.5%) patients with suspected IPN had organ failure, the majority of which had single organ failure, which was found in 52 (65%) cases. Amongst them, 36 (45%) had early-onset organ failure within the first week of the disease. Acute lung injury (ALI) alone existed in 43 (33.1%) cases, followed by a combination of ALI and acute kidney injury (AKI) in 14 (0.8%) patients, while AKI alone was present in nine (6.9%) cases. Cardiovascular system (CVS) organ failure was seen in four (3.1%) cases in association with ALI. The three-organ failures were found in 10 (7.7%) of patients. The mean time of resolution of ALI, AKI, and CVS organ failure was 9.48 ± 5.60 days, 9.62 ± 5.49 days, and 5.50 ± 2.38 days, respectively.

Comparing the relation between complications, including organ failure, infected necrosis, and death, with the extent of necrosis, we found that rates of complications were markedly greater in cases with a higher extent of pancreatic necrosis, with p-values <0.001, 0.004, and 0.032, respectively, for the aforementioned complications (Table 2).

**Table 2:** Association of extent of necrosis with organ failure, infected necrosis, and mortality *P-values <0.05 are considered significant.

Parameters	<30% necrosis	30%-50% necrosis	>50% necrosis,	p-value
Organ failure, n(%)	11 (13.8%)	33 (41.3%)	36 (45%)	<0.001*
Infected necrosis, n(%)	3 (5.6%)	20 (37.7%)	30 (56.6%)	0.004*
Mortality, n(%)	3 (10.7%)	11 (39.3%)	14 (50%)	0.032*

Among our study population, 88 (67.7%) patients required intervention, 66 (75%) of whom required PCD, 18 (20.5%) required endoscopic ultrasonography (EUS)-guided drainage (cystogastrostomy/cystoduodenostomy), and four (4.5%) required both PCD and EUS-guided drainage.

Evaluating the outcomes between the group of patients who needed intervention, which were 88 (67.7%) cases, and those who did not need intervention, which was 42 (32.3%) cases, we found that patients requiring intervention had markedly higher rates of organ failure, need for ICU, need for surgery, and death. The amount of pancreatic necrosis was also significantly higher in patients requiring intervention (p<0.001) (Table 3).

**Table 3:** Comparison of the need for intervention with various outcome parameters CTSI: computed tomography severity index; ICU: intensive care unit *P-values <0.05 are considered significant.

Variables	Non-intervention group (N=42)	Intervention group (N=88)	p-value
Organ failure, n (%)	20 (47.6%)	60 (68.18%)	0.024*
Need for ICU, n (%)	8 (19.04%)	47 (53.4%)	<0.001*
Need for surgery, n (%)	0 (0%)	18 (20.45%)	0.005*
Mortality, n (%)	2 (4.76%)	26 (29.54%)	0.001*
Pancreatic necrosis, n (%) (<30%, 30-50%, >50%)	24 (57.14%), 10 (23.81%), 8 (19.04%)	9 (10.22%), 37 (42.04%), 42 (47.72%)	<0.001*
CTSI score, n (%) (6, 8, 10)	12 (28.57%), 20 (47.6%), 10 (23.8%)	5 (5.6%), 28 (31.81%), 55 (62.5%)	<0.001*

Also, cases with an established diagnosis of infected pancreatic necrosis had significantly higher rates of organ failure, required multiple PCDs, and had higher amounts of pancreatic necrosis. The confirmed infected necrosis group also had higher rates of MDR infections, longer duration of hospital stays, need for ICU stay, ventilator requirement, higher need for surgery, and death compared to the sterile necrosis group (Table 4).

**Table 4:** Comparison of the existence of infected necrosis with various outcome parameters ICU: intensive care unit; MDRO: multidrug-resistant organisms; PCD: percutaneous catheter drain *P-values <0.05 are considered significant.

Variables	Sterile necrosis N=35	Infected necrosis N=53	p-value
Organ failure, n(%)	17 (48.6%)	43 (81.1%)	0.001*
Multiple (>2) PCD requirement, n(%)	5 (14.3%)	30 (56.6%)	0.001*
Amount of necrosis, n(%) (<30%, 30-50%, >50%)	9 (25.7%), 18 (51.4%), 8 (22.9%)	3 (5.7%), 20 (37.7%), 30 (56.6%)	0.001*
MDRO, n(%)	9 (25.7%)	35 (66%)	<0.001*
Need for ICU, n(%)	10 (28.6%)	37 (69.8%)	<0.001*
Need for ventilator support, n(%)	8 (22.9%)	27 (50.9%)	0.008*
Need for surgery, n(%)	3 (8.5%)	15 (28.3%)	0.002*
Length of hospital stay, mean±SD	16.51±7.55	22.55±10.35	0.008*
Length of ICU stay, median (IQR)	4.05 (2.80-5.45)	9.5 (7.42-11.58)	<0.001*
Mortality, n(%)	4 (11.4%)	16 (30.18%)	0.030*

Among 88 patients who underwent intervention, 53 (60.22%) patients grew organisms in the first culture. The most prevalent organism isolated was *E. coli* in 17 (32.07%) patients followed by *A. baumannii* in 12 (22.64%) patients. Additionally, over the entire course of the hospital stay, Gram-negative organisms were the most common in pancreatic (peri)site infections, with *E. coli *in 42 (30.88%) cases, followed by *A. baumannii* in 30 (22.05%) cases. Where the duration of illness was prolonged, the most common organism identified beyond the second week of illness was *A. baumannii* in 17 (32%) cases. There was also the emergence of Gram-positive organisms after the third week of illness (Appendix A).

Among 130 patients with suspected IPN, 42 (32.3%) patients had extrapancreatic infections (EPI). Overall, 107 pathogenic isolates were grown in the patients with suspected IPN from various extrapancreatic sites. The most common site of EPI was respiratory tract infections, where *Pseudomonas aeruginosa* (*P. aeruginosa*), followed by *K. pneumoniae*, were the most common organisms detected (Appendix B).

It is worth noting that positive fungal culture was found in six (3.4%) patients, among which four (66.67%) patients had fungal growth in urine and two (33.33%) patients had fungal growth in blood. The most common organism was *Candida albicans* (*C. albicans*) in five (83.33%) patients, and *Candida glabrratta* (*C. glabrratta*) species was found only in one (16.67%) patient.

**Figure 3:**
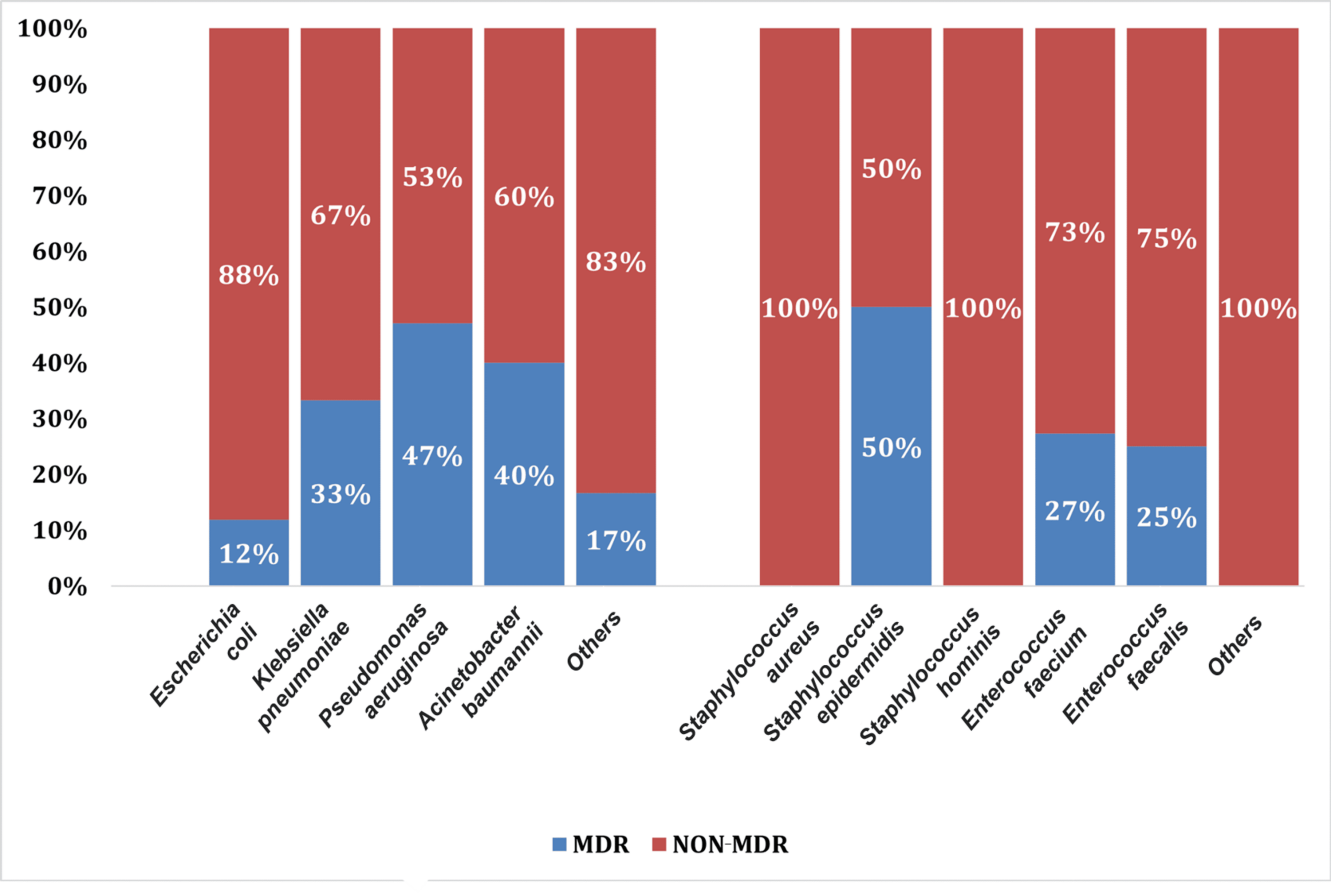
Multi-drug resistance (MDR) spectrum of extrapancreatic site infection

**Figure 4:**
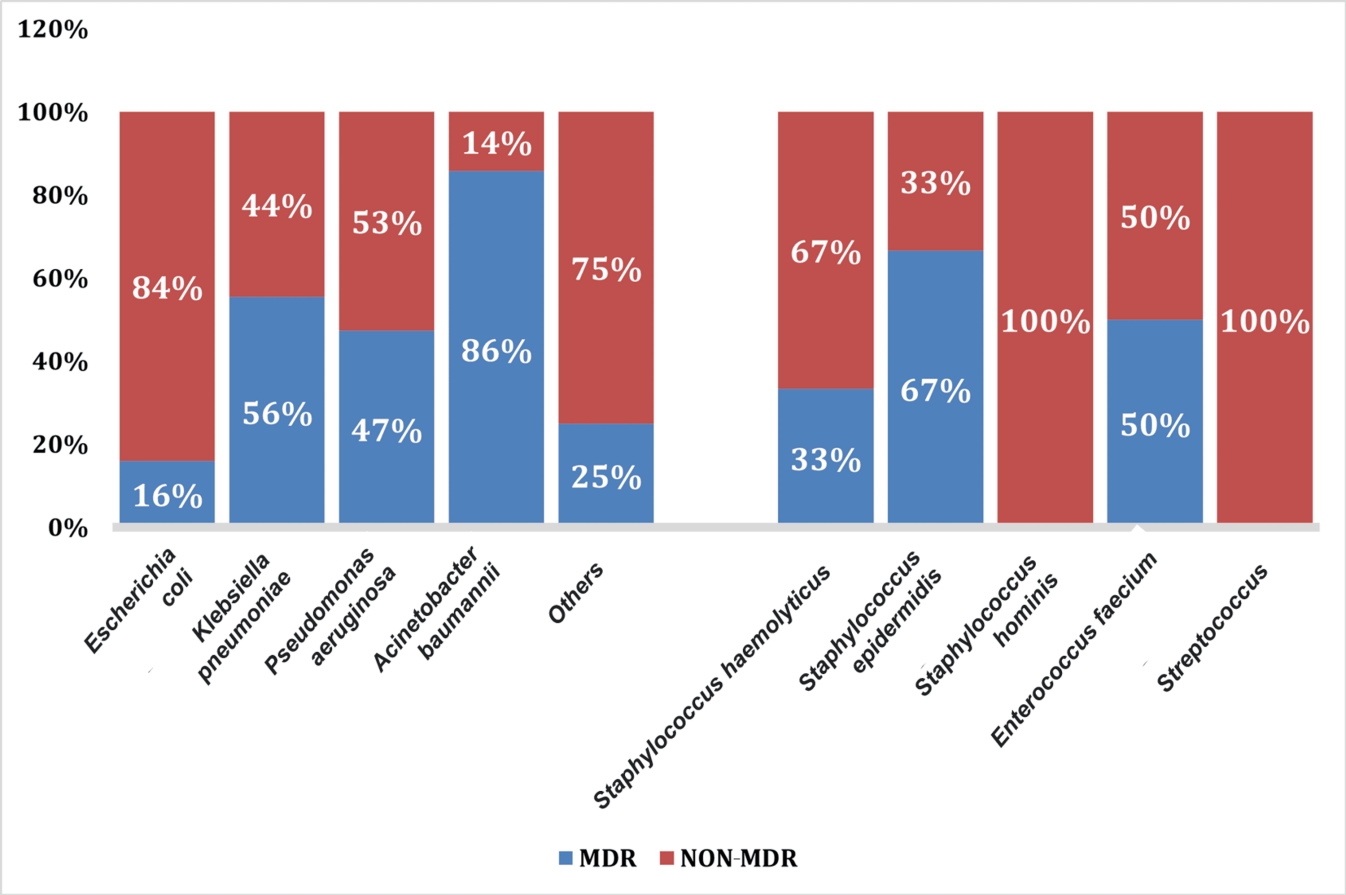
Multi-drug resistance (MDR) spectrum of Pancreati(peri) infection

Our study analyzed the drug resistance rates. The most common Gram-negative bacteria exhibited maximum resistance to ampicillin and third- and fourth-generation cephalosporins. The resistance rates of *E. coli *were most commonly found against quinolones (72%), followed by piperacillin-tazobactam (28%). However,* E. coli* did not exhibit any resistance to colistin. *Klebsiella pneumoniae* exhibited 40%-50% resistance to quinolones, followed by 38% resistance to piperacillin tazobactam. *Acinetobacter baumannii* and *P. aeruginosa* revealed maximum resistance to piperacillin-tazobactam (39% and 48%, respectively) and cefaperazone-sulbactam (41% and 52%, respectively) amongst Gram-negative organisms. *Pseudomonas aeruginosa* showed maximum resistance to carbapenems (51%-60% resistance rate) and meropenem (60% resistance rate). It was evident that *A. baumannii* and *P. aeruginosa *revealed maximum resistance to most of the drugs (Figures 3, 4).

In our study, 38 (64.4%) patients of 59 patients who were administered prior antibiotics received them during the first seven days of illness. These cases had significantly higher rates of development of MDR organisms (p<0.001), fungal infections (p = 0.012), and combined pancreatic and extrapancreatic infections (p = 0.001). However, prior antibiotic exposure did not affect the development of infection of pancreatic (peri) necrosis and extrapancreatic infections (Table 5).

Prior antibiotic exposure during the first seven days of disease was also linked to a greater need for ICU support (p = 0.002) and death (p = 0.018). There was a trend towards a higher need for surgery in cases who received empirical antibiotics in the first week, although it did not achieve statistical significance (p = 0.066). Yet, there was no statistically significant variation in the need for intervention between these two groups (Table 5).

**Table 5:** Comparison between prior antibiotic therapy during the first week of illness and no prior antibiotics with infectious complications ICU: intensive care unit; MDR: multi-drug resistance *P-values <0.05 are considered significant.

Parameters	Patients with prior antibiotic exposure in the first week (N=38)	Patients with no prior antibiotic exposure in the first week (N=92)	p-value
Infected necrosis (pancreatic(peri) infection), n(%)	15 (39.47%)	24 (26.08%)	0.128
Extrapancreatic infections, n(%)	8 (21.05%)	12 (13.04%)	0.164
Combined infection, n(%)	19 (50%)	17 (18.47%)	0.001*
MDR organisms, n(%)	24 (63.15%)	22 (23.91%)	<0.001*
Fungal infection, n(%)	4 (10.52%)	2 (2.1%)	0.012*
Need for intervention, n (%)	26(68.42%)	62(67.3%)	0.254
Need for surgery, n (%)	10(26.3%)	8(8.6%)	0.066*
Need for ICU, n (%)	25(65.78%)	30(32.60%)	0.002*
Mortality, n (%)	15(39.47%)	13(14.13%)	0.018*

## Discussion

Demographics

Acute necrotizing pancreatitis is a severe form of AP and is defined as the necrosis of the pancreatic parenchyma with or without necrosis of peripancreatic tissue [3]. About 20%-30% of AP cases develop necrotizing pancreatitis, which is linked to higher morbidity and death rates [8, 12]. Infected pancreatic necrosis is suspected when a patient with necrotizing pancreatitis develops persistent clinical manifestations of sepsis, i.e., fever of >38˚C beyond the first seven days of disease along with leukocytosis and deterioration in clinical condition [13].

In our study, 130 patients with suspected IPN were enrolled, and their natural history was evaluated. The mean age of the patients was 39.75 years, with 95 (73.9%) males and 35 (26.1%) females. An epidemiological study from Taiwan also showed similar demographic characteristics, whereas another one from Japan showed a higher mean age and male-to-female ratio of 2:1 [14, 15]. A systematic review showed no statistically significant difference between men and women [1]. Male predominance in our study could be explained by alcohol as a cause of pancreatitis, which was the most common aetiology in our study. The distribution of aetiologies was comparable to data from Asia [14, 15]. In Western countries, gallstone disease is the most prevalent cause because of the higher prevalence of obesity [16].

The median time taken from the onset of pain to first medical contact in our study cohort was three days, and the median time taken to admission was nine days, suggesting that infectious complications set in beyond one week of pain. The patients with suspected IPN enrolled in our study underwent a CECT scan of the abdomen. The most common type of necrosis was in the form of combined pancreatic and extrapancreatic necrosis in 111 (85.4%) patients, and around 51 (39.2%) patients had vascular complications. The mean CTSI score was 8.72±1.43. A retrospective study of 100 AP patients by Mortele et al. also showed similar results, with a mean CTSI score of 9.4 [17]. The high CTSI score in our study could be explained by the fact that all cases had either moderately severe or severe AP. 

The extent of necrosis

In the current study, 36 (27.7%) patients had <30% necrosis, 47 (36.2%) had 30%-50% necrosis, and 47 (36.2%) cases had >50% necrosis. We found a strong correlation between the extent of pancreatic necrosis and the development of organ failure, infection of the necrosis, and mortality. Studies by Garg et al. and Isenmann et al. revealed that the extent of necrosis was significantly associated with the development of organ failure and mortality [18, 19]. Garg and colleagues suggested that there was a 78% probability of developing organ failure in cases with >50% necrosis [18]. However, the investigations by Beger et al. and Tenner et al. were not able to reveal the association between the incidence of organ failure and the extent of necrosis, most probably due to their small sample sizes [20, 21]. 

The magnitude of the initial pancreatic injury determines the extent of necrosis and the severity of the inflammatory response. The release of cytokines and vasoactive peptides, along with the activation of inflammatory cells, is what causes this reaction. Organ failure and systemic insults are the results of these events. The inflammatory cascade is further enhanced when the pancreatic necrotic tissue becomes infected [18].

Organ failure

Among 76 cases with persistent organ failure, 32 (42.1%) patients developed organ failure within the first week of illness. After the first week, patients with early organ failure did not exhibit significantly increased mortality rates. This is consistent with conclusions from previous studies, which suggested that the timing of the onset of organ failure was not associated with mortality [22]. Acute lung injury alone was present in 43 (33.1%) cases, followed by a combination of ALI and AKI in 14 (0.8%) patients. A previous study by Guo et al. also showed the same extrapancreatic results as ours [23]. Another study by Machicado et al. also showed that the most significant factor influencing death is persistent organ failure. They also found that multiple persistent organ failure is linked to higher death rates, which is concordant with our study results [24].

Intervention

Out of 130 patients with suspected IPN in our study group, 88 (67.7%) patients required intervention in one or the other form and 42 (32.3%) patients were managed conservatively. The median interval between the onset of pain and the first PCD insertion was 18 days. The median duration of all PCDs in our study was found to be 35 days. Among the 88 (67.7%) patients who required intervention, 70 (79.5%) required PCD, and 18 (20.45%) required EUS-guided drainage. The study showed that 44 (62.8%) patients out of 70 patients treated with PCD could be managed successfully with PCD alone. Amongst the remaining patients who could not be managed successfully with PCD, 18 (25.7%) patients underwent surgical necrosectomy, and four (5.7%) patients died. Multiple previous studies showed that patients could be successfully managed with PCD alone [25-27]. A large prospective study on 639 patients with ANP concluded that delayed intervention instead of surgical necrosectomy could enhance results in cases with IPN [28]. These results from our study thus indicate that PCD facilitated the clearance of pancreatic collection/necrosis and infectious debris, hence effectively controlling the inflammatory cascade, which might help delay or even eliminate the need for surgical necrosectomy.

Microbiological profile

Pancreatic/Peripancreatic Site Infection

In the current study, Gram-negative organisms were the most prevalent isolates from infected necrosis. The most prevalent organism isolated was *E. coli* (30.18%), followed by *A. baumanni*i (22.64%). Similar results were found in previous literature where Gram-negative organisms were most common among patients with pancreatitis [29-31]. However, *A. baumannii* was the most common Gram-negative organism in the investigation by Hao et al. [29], and *K. pneumoniae* was the most common among patients of the study by Biberci et al. [30], whereas in concordance with our results, E. coli was the commonest in the study by Lu et al. [31]. On the contrary, Jiang et al. found Gram-positive organisms to be the most common isolates of pancreatic infection [32].

Extrapancreatic Site Infections

In our cases, the common organisms from extrapancreatic sites were *P. aeruginosa* and *A. baumannii*. In the first two weeks of AP, bloodstream infection is the commonest, whereas respiratory tract infections begin to increase beyond the third week. Similarly, several other Asian and European studies from various tertiary care centres showed that the incidence of EPI is increasing, with respiratory tract and bloodstream infections as the common sites [31-33]. Hence, the results from our study and several other studies quoted above suggest that the increasing incidence of EPI might be explained by increased nosocomial and healthcare-associated disease burden. 

Drug Resistance Pattern

We found the drug resistance of pathogens causing pancreatic/peripancreatic infection to be alarmingly high. This alarming increase in MDR could be clarified by the irrational usage of prophylactic antibiotics. Our investigation showed that there was a significant association between prophylactic antibiotic therapy during the first week of illness and the development of multi-drug-resistant organisms (MDRO). Multi-drug resistance infection was associated with an increased need for an ICU stay and prolonged hospital admission compared to non-MDR infections. This, in turn, led to the development of nosocomial infections with a further increase in MDR strains. Multi-drug resistance infection was also associated with higher rates of mortality. Similarly, an expert review on anti-infective therapy and a recent European study suggested that prophylactic antibiotic therapy was associated with an increase in the emergence of MDR strains [34, 35]. A recent Indian multicenter study on antibiotic use in acute pancreatitis patients showed that 66.4% of patients received prophylactic antibiotics, which led to the development of MDR infections [36].

Limitations

This is a single-centre study. Since ours is a tertiary care academic centre with vast experience in managing patients with AP, our results cannot be extrapolated to other healthcare settings.

Since the majority of pancreatitis cases in our investigation were referred from other healthcare settings, the time of infection development, their source, drug resistance patterns, management, and prognosis may be altered. However, this gives us the ability to analyze real-world data of AP patients with suspected infected necrosis being referred to higher centres.

We did not have a control group of AP cases that were not suspected of having infected pancreatic necrosis for comparison in this study.

We did not perform FNA of necrotic tissue to confirm the presence of infection. We labelled cultures that did not grow any isolates repeatedly as sterile. However, we acknowledge that the inability to grow on cultures may not be synonymous with sterile collections.

## Conclusions

Thus, we conclude that infected pancreatic necrosis is linked to high rates of morbidity and death. The irrational usage of prophylactic antibiotic therapy in cases with suspected infected pancreatic necrosis alters their natural course and makes them vulnerable to combined pancreatic and extrapancreatic infections with a higher MDR incidence, leading to increased morbidities and mortalities. This study is an eye-opener regarding the alarming increase in the incidence of MDR organisms in cases with infected pancreatic necrosis. This, in turn, is increasing healthcare expenditure in an already resource-constrained country. The growth of MDR organisms is further worsening AP-related morbidity and mortality. There is a need to develop evidence-based recommendations and guidelines regarding appropriate indication, timing, and duration of antibiotic use amongst AP patients with suspected IPN. Awareness needs to be created amongst healthcare professionals regarding the need to give targeted antimicrobials rather than broad-spectrum high-end antibiotics for all pancreatic infections and the need to scale them down as and when the culprit organism with its antibiotic sensitivity pattern is identified. This study is a ground for future research in this difficult-to-treat category of AP cases with infected pancreatic necrosis.

## Appendices

Appendix A

**Table 6:** Timing and composition of pancreatic(peri) infection A.baumannii: acinetobacter baumannii; E.coli: escherichia coli; K.pneumoniae: Klebsiella pneumoniae

Week of illness	Organism	Number of isolates
2nd	E.coli	20
	K.pneumoniae	6
	Pseudomonas aeruginosa	4
	A.baumannii	2
	Enterococcus faecium	3
3rd	E.coli	12
	K.pneumoniae	10
	Pseudomonas aeruginosa	6
	A.baumannii	12
	Enterococcus faecium	4
	Enterococcus faecalis	2
	Staphylococcus hominis	2
	Proteus mirabilis	2
≥4	E.coli	10
	K.pneumoniae	5
	Pseudomonas aeruginosa	7
	A.baumannii	17
	Enterococcus faecium	4
	Enterococcus faecalis	5
	Staphylococcus epidermidis	2
	Staphylococcus aureus	1
	Proteus mirabilis	2

Appendix B

**Table 7:** Site and pathogenic composition of extrapancreatic site infections

Organism	Bloodstream		Respiratory tract		Urinary tract	Ascitic fluid	Pleural fluid
	Peripheral line	Central line	Sputum	ET aspirate			
Escherichia coli, n(%)	2 (6.06%)	0 (0%)	1 (2.7%)	2(5.5%)	5 (83.3%)	7 (30.4%)	2 (13.3%)
Acinetobacter baumannii, n(%)	6 (18.18%)	2 (6.06%)	4 (11.1%)	4 (11.1%)	0 (0%)	0 (0%)	7 (46.7%)
Klebsiella pneumoniae, n(%)	1 (3.03%)	2 (6.06%)	5 (13.8%)	4 (11.1%)	0 (0%)	4 (17.3%)	4 (26.7%)
Pseudomonas aeruginosa, n(%)	1 (3.03%)	1 (3.03%)	7 (19.4%)	5 (13.8%)	1 (16.7%)	10 (43.5%)	1 (6.7%)
Enterococcus faecium, n(%)	2 (6.06%)	0 (0%)	0 (0%)	0 (0%)	0 (0%)	0 (0%)	0 (0%)
Staphylococcus hominis, n(%)	3 (9.09%)	2 (6.06%)	0 (0%)	0 (0%)	0 (0%)	0 (0%)	0 (0%)
Staphylococcus epidermidis, n(%)	3 (9.09%)	0 (0%)	0 (0%)	0 (0%)	0 (0%)	0 (0%)	0 (0%)
Staphylococcus haemolyticus, n(%)	7 (21.2%)	0 (0%)	0 (0%)	0 (0%)	0 (0%)	1(0.8)	0 (0%)
Stenotrophomonas, n(%)	0 (0%)	0 (0%)	0 (0%)	0 (0%)	0 (0%)	0 (0%)	1 (6.7%)
Burkholderia cepacia, n(%)	1 (3.03%)	0 (0%)	0 (0%)	0 (0%)	0 (0%)	0 (0%)	0 (0%)
Citrobacter, n(%)	0 (0%)	0 (0%)	0 (0%)	0 (0%)	0 (0%)	1(0.8)	0 (0%)
Streptococcus pneumoniae, n(%)	0 (0%)	0 (0%)	2 (5.5%)	1 (2.7%)	0 (0%)	0 (0%)	0 (0%)
Providencia species, n(%)	0 (0%)	0 (0%)	0 (0%)	1 (2.7%)	0 (0%)	0 (0%)	0 (0%)
Total strains isolated	26	7	19	17	6	23	15
